# Five-Year Retrospective Analysis of Traumatic and Non-Traumatic Pneumothorax in 2797 Patients

**DOI:** 10.3390/healthcare13141660

**Published:** 2025-07-10

**Authors:** Ayhan Tabur, Alper Tabur

**Affiliations:** 1 Department of Emergency Medicine, SBÜ Gazi Yaşargil Training and Research Hospital, Diyarbakır 21010, Turkey; 2 Department of Thoracic Surgery, SBÜ Kocaeli City Hospital, Kocaeli 41001, Turkey; drtabur01@gmail.com

**Keywords:** pneumothorax, emergency medicine, spontaneous pneumothorax, traumatic pneumothorax, age-specific management

## Abstract

**Objectives:** Pneumothorax is a critical condition frequently encountered in emergency departments (EDs), with spontaneous pneumothorax (SP) and traumatic pneumothorax (TP) presenting distinct clinical challenges. This study aimed to evaluate the epidemiological characteristics, clinical outcomes, and treatment strategies for SP and TP across different age groups and provide insights for optimizing emergency management protocols. **Methods:** This retrospective cohort study analyzed 2797 cases of pneumothorax over five years (2018–2023) at a tertiary care center. Patients were stratified by age (18–39, 40–64, and >65 years) and pneumothorax type (SP vs. TP). Data on demographics, clinical presentation, treatment, hospital stay, recurrence, and complications were extracted from medical records. Comparative statistical analyses were also conducted. **Results:** The mean age of patients with SP was 32.5 ± 14.7 years, whereas patients with TP were older (37.8 ± 16.2 years, *p* < 0.001). Male predominance was observed in both groups: 2085 (87.0%) in the SP group and 368 (92.0%) in the TP group (*p* = 0.01). The right lung was more frequently affected in the SP (64.2%) and TP (56.0%) groups (*p* < 0.001). Age-related differences were evident in both groups of patients. In the SP group, younger patients (18–39 years) represented the majority of cases, whereas older patients (≥65 years) were more likely to present with SSP and required more invasive management (*p* < 0.01). In the TP group, younger patients often had pneumothorax due to high-energy trauma, whereas older individuals developed pneumothorax due to falls or iatrogenic causes (*p* < 0.01). SP predominantly affected younger patients, with a history of smoking and male predominance associated with younger age (*p* < 0.01). TP is more frequent in older patients, often because of falls or iatrogenic injuries. Management strategies varied by age group; younger patients were often managed conservatively, whereas older patients underwent more invasive procedures (*p* < 0.01). Surgical intervention was more common in younger patients in the TP group, whereas conservative management was more frequent in elderly patients (*p* < 0.01). The clinical outcomes differed significantly, with older patients having longer hospital stays and higher rates of persistent air leaks (*p* < 0.01). Recurrence was more common in younger patients with SP, whereas TP recurrence rates were lower across all age groups (*p* < 0.01). No significant differences were observed in re-expansion pulmonary edema, empyema, or mortality rates between the age groups, suggesting that age alone was not an independent predictor of these complications when adjusted for pneumothorax severity and management strategy (*p* = 0.22). **Conclusions:** Age, pneumothorax subtype, and underlying pulmonary comorbidities were identified as key predictors of clinical outcomes. Advanced age, secondary spontaneous pneumothorax, and COPD were independently associated with recurrence, prolonged hospitalization, and in-hospital mortality, respectively. These findings highlight the need for risk-adapted management strategies to improve triaging and treatment decisions for spontaneous and traumatic pneumothorax.

## 1. Introduction

Pneumothorax is a critical condition frequently encountered in the emergency department (ED), with an estimated annual incidence of 6–66 per 100,000 individuals and a recurrence rate of approximately 29% within the first year of resolution [1,2]. It is more prevalent in males and demonstrates a bimodal age distribution, with peak incidence in individuals aged 15–30 and >65 years [3,4]. Pneumothorax is broadly classified as spontaneous pneumothorax (SP) or traumatic pneumothorax (TP), the latter resulting from blunt or penetrating trauma or iatrogenic causes, such as central venous catheterization, mechanical ventilation, or thoracic procedures [1,2].

Spontaneous pneumothorax is further categorized into primary spontaneous pneumothorax (PSP), which occurs in patients without known lung disease, and secondary spontaneous pneumothorax (SSP), which is associated with underlying pulmonary pathologies such as chronic obstructive pulmonary disease (COPD), asthma, cystic fibrosis, interstitial lung disease, tuberculosis, and malignancy [5,6]. PSP typically affects young, thin males with a history of smoking, whereas SSP predominantly occurs in older individuals with significant pulmonary comorbidities [4,7,8].

The clinical presentation of pneumothorax varies widely, ranging from asymptomatic cases detected incidentally on imaging to life-threatening respiratory distress in cases of large or tension pneumothorax. The most commonly reported symptoms include dyspnea (45–93%), pleuritic chest pain (17–92%), and cough (20–43%), whereas hypoxia (SpO_2_ < 90%) is relatively uncommon, occurring in only 3–11% of cases [3,8]. Physical examination findings, such as unilateral diminished breath sounds (73–81%), reduced tactile fremitus, and hyperresonance on percussion, can aid in the diagnosis [9]. The emergency management of pneumothorax is guided by the patient’s clinical stability, size, and underlying lung function. Hemodynamically unstable patients with suspected tension pneumothorax require immediate needle decompression, followed by definitive chest tube thoracostomy [10]. For stable patients, management ranges from conservative observation with high-flow oxygen therapy to needle aspiration, chest tube drainage, or surgical intervention, particularly in cases of large, recurrent, or persistent pneumothorax [6,11,12]. Spontaneous and traumatic pneumothoraxes differ significantly in terms of etiology and underlying pathophysiology. However, from a clinical perspective, both present acutely to emergency services and require rapid intervention. Understanding their comparative epidemiology, particularly across age groups, may provide critical insights into tailoring emergency management protocols and improving outcomes. This distinction is increasingly relevant, given the growing proportion of iatrogenic and geriatric trauma-related pneumothorax presentations. Age has a substantial impact on the clinical manifestations, underlying etiology, and treatment response of pneumothorax. Younger individuals frequently present with primary spontaneous pneumothorax, often associated with subpleural blebs and smoking, whereas older patients tend to exhibit secondary pneumothorax due to chronic pulmonary disease or trauma. Therefore, an age-stratified analysis allows for a more granular understanding of pneumothorax presentation and management and aligns with emerging recommendations for age-adapted care pathways in emergency medicine. Understanding pneumothorax patterns in a population can inform clinical practice and improve patient outcomes.

This retrospective observational study aimed to evaluate the epidemiological characteristics, treatment strategies, and clinical outcomes of traumatic and spontaneous pneumothorax in a tertiary care setting in a large cohort of 2797 patients over five years. By analyzing the differences in presentation, management, and prognosis, this study sought to provide insights for optimizing emergency management protocols through descriptive and comparative epidemiological assessments.

## 2. Material and Methods

### 2.1. The Study Design and Setting

This study was conducted as a single-center retrospective observational study examining pneumothorax cases over a five-year period (April 2018–April 2023). This study was conducted at the Diyarbakır Gazi Yaşargil Education and Research Hospital, a large tertiary referral center in Turkey.

### 2.2. The Inclusion and Exclusion Criteria

All patients diagnosed with pneumothorax (spontaneous or traumatic) during the study period were included, regardless of age or sex. Cases were identified through the hospital’s electronic medical record system using ICD-10 diagnostic codes (J93.x series). Inpatient and outpatient visits were also screened. Patients were included irrespective of their management approach (conservative versus interventional).

Patients treated outside the study period and those with incomplete medical records (missing demographic or outcome data) were excluded. If multiple episodes of pneumothorax were recorded in the same patient, each episode was treated as a separate event, acknowledging the possibility of recurrence in the same patient.

## 3. The Data Collection

Patient data were retrieved from hospital information systems and medical records using a standardized data collection form. The recorded variables included demographic characteristics (age, sex, and pneumothorax classification) that distinguished between spontaneous and traumatic pneumothoraxes. SP classification was performed to determine whether SSP was present due to an underlying lung disease. The pneumothorax side was noted as left, right, or bilateral, and the presentation type was categorized as emergency, outpatient, or inpatient presentation. Comorbidities were extracted to calculate the Charlson Comorbidity Index (CCI) when sufficient diagnostic codes were present. In cases of TP, the etiology was specified as blunt trauma, penetrating trauma, or iatrogenic (post-procedure complications resulting from central venous catheter placement or mechanical ventilation). High-energy trauma was defined as an injury involving high-velocity motor vehicle accidents, falls from heights >3 m, or crush injuries. The initial clinical evaluation and management of pneumothorax in the ED were performed under the supervision of board-certified emergency medicine specialists. Therefore, physician-level variability was minimal, and no further subgroup analyses by provider specialty were conducted.

The treatment approach for each case was documented to determine whether the patient underwent observation alone, needle aspiration, chest tube thoracostomy, or surgical intervention. In cases requiring surgery, the procedure was classified as either video-assisted thoracoscopic surgery (VATS) or open thoracotomy with bleb resection and pleurodesis. The admitting department (thoracic surgery, emergency medicine, or intensive care unit) was recorded, as it often correlated with the chosen management strategy.

Clinical outcomes were systematically evaluated, including length of hospital stay (measured in days for admitted cases), complications, in-hospital mortality, and recurrence. Complications were defined as persistent air leaks lasting longer than five days, re-expansion pulmonary edema, or empyema. Recurrence was defined as a new pneumothorax occurring on the same side following initial successful treatment at any time during the study period. Recurrence events were primarily identified through hospital records; however, underreporting may have occurred if patients sought medical attention at other facilities.

PSP and SSP were diagnosed based on radiological findings and clinical histories. High-resolution computed tomography (HRCT) was performed in patients with atypical presentations or suspected underlying lung diseases. All TP cases were categorized based on the trauma mechanism (blunt, penetrating, or iatrogenic), as confirmed by the emergency records and imaging.

### Ethical Consideration

Ethical approval was obtained from the Institutional Ethics Committee (Approval No. 2023/47), and the requirement for informed consent was waived because of the retrospective nature of this study. Patient confidentiality was ensured in accordance with the principles of the Declaration of Helsinki.

## 4. The Statistical Analysis

Descriptive and inferential statistical methods were used for data analysis. Continuous variables were summarized as mean ± standard deviation (SD) or median with interquartile range (IQR), depending on the distribution of the data. Normality was assessed using the Kolmogorov–Smirnov test. Categorical variables are reported as frequencies and percentages. Comparisons between the TP and SP cases were conducted using the chi-square test for categorical variables, Student’s *t*-test for normally distributed continuous variables, and the Mann–Whitney U test for non-normally distributed continuous variables. Multivariate logistic regression analyses were performed to identify the independent predictors of persistent air leaks, recurrence, and in-hospital mortality. The variables included in the models were age, sex, pneumothorax type, presence of underlying lung disease, smoking history, and initial management strategy. Kaplan–Meier survival curves and Cox proportional hazards models were used for mortality analysis. Mixed-effects logistic regression models were applied to account for repeated events within individuals in the recurrence analysis.

All statistical analyses were performed using SPSS version 26 (IBM Corp., Armonk, NY, USA), and *p*-values < 0.05 were considered statistically significant.

## 5. Results

Over the five-year study period, 2797 episodes of pneumothorax were identified. A total of 839 (30 %) cases were unique. The remaining 1958 patients (70%) included individuals with recurrent pneumothorax or bilateral involvement, which were counted as separate events. Among all cases, 2397 (85.7%) were classified as non-traumatic pneumothorax and 400 (14.3%) as traumatic pneumothoraxes. PSP was diagnosed in 1558 patients (55.7%), and SSP was identified in 839 patients (30.0%). Among traumatic pneumothorax, 220 cases (7.9%) resulted from blunt trauma, 120 (4.3%) from penetrating trauma, and 60 (2.1%) from iatrogenic pneumothorax. A male predominance (85.0%) was observed in 2377 cases. A history of smoking was documented in 1818 patients (65.0%). Among the study population, 559 (20.0%) had a history of COPD diagnosis. The anatomical distribution of pneumothorax showed bilateral pneumothorax in 84 (3.0%) patients. The highest proportion of cases was observed in young adults aged 18–24 years old, accounting for 838 cases (30.0%), whereas the lowest proportion was observed in those aged 65 years old and older, comprising 223 cases (8.0%). Table 1 shows the baseline characteristics of the study population.

The mean age of patients with SP was 32.5 ± 14.7 years, whereas those with TP were significantly older (37.8 ± 16.2 years; *p* < 0.001). Males predominated in both groups: 2085 (87.0%) spontaneous and 368 (92.0%) traumatic cases (*p* = 0.01). Pneumothorax affected the right lung in 1538 (64.2%) spontaneous and 224 (56.0%) traumatic cases (*p* < 0.001). Left-sided pneumothorax occurred in 1175 (49.0%) spontaneous and 160 (40.0%) traumatic cases (*p* < 0.001). A history of lung disease was more common in the SP group (839 patients, 35.0%) than in the traumatic group (20 patients, 5.0%) (*p* < 0.001). A history of smoking was more prevalent in patients with spontaneous pneumothorax (1818 cases, 65.0%) than in those with traumatic pneumothorax (192 cases, 48.0%) (*p* < 0.001).

Initial noninvasive observations were performed in 1438 (60.0%) spontaneous cases (*p* < 0.001), and hospital admission was required for 340 (85.0%) traumatic cases (*p* < 0.001).

Chest tube placement was the most common intervention, performed in 2061 (86.0%) spontaneous and 392 (98.0%) traumatic cases (*p* < 0.001). Surgical intervention was required in 240 (10.0%) spontaneous and 80 (20.0%) traumatic cases (*p* < 0.001). Ambulatory and conservative management was more frequent in patients with spontaneous (360, 15.0%) than traumatic pneumothorax (28, 7.0%) (*p* < 0.001).

The mean hospital stay was longer for TP (5.9 ± 3.4 days, *p* < 0.001). Persistent air leaks were more frequent in traumatic cases (28 cases, 7.0%) (*p* < 0.001). The recurrence rate was higher in the SP group (431 cases, 18.0%) (*p* < 0.001). A detailed comparison between the SP and TP patients is presented in Table 2.

In patients with SP, a male predominance was observed across all age groups (*p* < 0.01), with the highest proportion in the 18–39 age group (55.01%). A history of smoking was significantly more prevalent among younger patients (*p* < 0.01). Younger patients were more likely to be managed using noninvasive observation (*p* < 0.01), whereas the hospital admission and chest tube placement rates increased with age (*p* < 0.01). Surgical intervention was significantly more frequent in older patients (*p* < 0.01). The length of hospital stay was significantly longer in patients aged >65 years (5.2 ± 1.8 days) than in younger groups (*p* < 0.01). Persistent air leaks were more common, and recurrence rates were significantly higher in older patients (*p* < 0.01).

In patients with TP, male predominance remained high across all age groups (*p* < 0.01). A history of smoking was significantly associated with younger age (*p* < 0.01). Hospital admission rates were significantly higher in all age groups (*p* < 0.01). Chest tube placement was the predominant intervention, particularly in the elderly group (*p* < 0.01). Surgical interventions were more frequent in younger patients (*p* < 0.01), whereas conservative management was more common in older patients (*p* < 0.01). The length of hospital stay was significantly longer in older patients than in younger patients (*p* < 0.01). No significant differences were observed in re-expansion pulmonary edema (*p* = 0.22), empyema (*p* = 0.22), or mortality (*p* = 0.22). Recurrence was significantly more frequent in younger patients (*p* < 0.01). Table 3 presents a comparative analysis of the demographic characteristics, management strategies, and clinical outcomes of SP and TP across age groups (18–39, 40–64, and >65 years).

Multivariate logistic regression analyses were conducted to determine the independent predictors of five key clinical outcomes: persistent air leaks, surgical intervention, recurrence, prolonged hospital stay (defined as a length of stay ≥5 days), and in-hospital mortality. Age ≥ 65 years was found to be a consistent risk factor across multiple outcomes, significantly increasing the likelihood of recurrence (OR: 1.92, 95% CI: 1.20–3.07, *p* = 0.006), prolonged hospitalization (OR: 3.52, 95% CI: 2.11–5.87, *p* < 0.001), and in-hospital mortality (OR: 3.92, 95% CI: 1.90–8.01, *p* < 0.001). SSP was strongly associated with persistent air leaks (odds ratio [OR], 2.90; *p* = 0.003), surgical intervention (OR, 1.88; *p* = 0.018), and an increased risk of mortality (OR, 2.72; *p* = 0.002). Smoking was independently associated with recurrence (OR, 1.51; *p* = 0.013) and prolonged hospital stay (OR, 1.65; *p* = 0.004) but did not significantly affect mortality or persistent air leaks. COPD emerged as a strong predictor of multiple adverse outcomes, including persistent air leaks (OR, 2.14; *p* = 0.003), recurrence (OR, 1.49; *p* = 0.045), prolonged hospitalization (OR: 2.78, *p* < 0.001), and mortality (OR: 2.61, *p* = 0.004) (Table 4).

**Figure 1:**
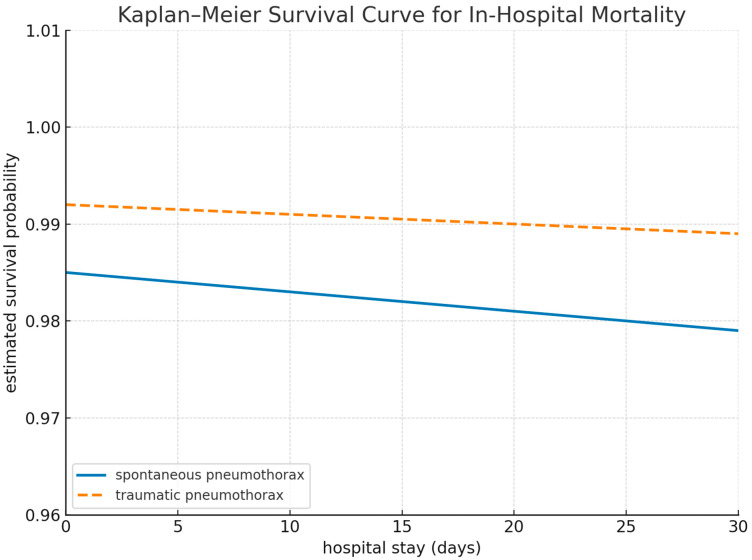
Kaplan–Meier survival curves comparing the in-hospital survival probabilities between patients with spontaneous and traumatic pneumothorax over a 30-day period.

Kaplan–Meier survival analysis was conducted to compare in-hospital mortality between patients with SP and TP. Although the overall number of deaths was low in both groups, the probability of survival declined more rapidly in the SP group, particularly within the first 10 days of hospitalization. On day 10, the estimated survival probability was 0.984 in the SP group and 0.994 in the TP group, respectively. The 30-day in-hospital mortality rates were 1.5% (362,397) for SP and 0.8% (3400) for TP. The log-rank test did not reveal a statistically significant difference between the groups (*p* = 0.24), although the numerical trend favored better survival in the TP group (Figure 1).

## 6. Discussion

This study comprehensively analyzed the demographic characteristics, treatment strategies, and clinical outcomes of SP and TP across different age groups. These findings confirm age-related variations in the presentation and management of pneumothorax, which have significant implications for emergency medical decision-making and patient prognosis.

Male predominance was observed in both SP and TP across all age groups, consistent with the findings of Weissberg et al. and Nishizawa et al. [10,13]. A history of smoking is more frequently reported in younger patients with SP, reinforcing its role as a major risk factor [14]. In contrast, older patients with SP exhibited higher rates of SSP, likely due to the increased prevalence of chronic pulmonary diseases such as COPD and interstitial lung disease [5,15].

In patients with TP, younger patients were more frequently affected by high-energy trauma mechanisms, whereas elderly patients experienced pneumothorax secondary to falls, iatrogenic causes, or rib fractures associated with osteoporosis, which was similar to the findings of Ishak et al. [16]. The etiological patterns of TP differed significantly between age groups, emphasizing the need for age-specific diagnostic and management strategies in the ED setting.

Significant differences in treatment approaches were observed between the age groups for both the SP and TP. In SP, younger patients were more likely to be managed conservatively, whereas older patients underwent chest tube placement or surgical interventions more frequently, which is consistent with previous reports by Mehrabi et al. and Erez et al. [17,18]. Higher rates of persistent air leaks and prolonged hospital stays in elderly SP patients have been well documented, justifying an earlier surgical intervention in this population.

In the TP group, chest tube placement was the predominant intervention in all age groups. However, surgical interventions were more frequently performed in younger patients, whereas conservative management was more often applied in elderly patients, which is in agreement with the findings of Gerhardy et al. [19,20]. The preference for non-surgical approaches in elderly TP patients may be explained by their higher rates of comorbidities, frailty, and limited physiological reserves, making invasive procedures less favorable [5].

Age was a significant determinant of clinical outcomes, including length of hospital stay, recurrence rates, and complications. Older patients with SP and TP experience prolonged hospital stays, a trend that has been linked to delayed recovery, increased complication rates, and underlying comorbidities [10].

The recurrence rate in patients with SP was significantly higher than that in patients with TP, with younger patients with SP particularly affected. These findings reinforce the well-documented risk of recurrent pneumothorax in young, tall, and thin males with subpleural blebs, as described by Hallifax et al. [1]. In contrast, the recurrence rates in TP are lower, especially in older patients, potentially because of their lower long-term survival and reduced physical activity levels, which may decrease re-exposure to trauma-related injuries [1,2,21].

Persistent air leaks were more frequently observed in older SP and TP patients, likely due to age-related reductions in pleural healing capacity and a higher prevalence of underlying lung disease [12]. In contrast, no significant differences were observed in re-expansion pulmonary edema, empyema, or mortality rates between the age groups, suggesting that age alone is not an independent predictor of these complications when adjusted for pneumothorax severity and management strategy [22].

The observed age-related differences in pneumothorax management and outcomes highlight the importance of age-specific treatment algorithms in the EDs. Based on the findings of this study, several key recommendations can be made: young SP patients (<40 years) with small, first-episode pneumothorax should be considered for ambulatory conservative management, a strategy increasingly supported by studies favoring outpatient observation models [19,20]. Older patients with SP (>65 years) and those with SSP should be classified as high-risk for prolonged hospitalization, recurrence, and persistent air leaks, justifying early intervention with pleurodesis or surgical management [14]. Elderly patients with TP should not automatically undergo aggressive interventions, as conservative management is frequently applied and often successful. However, a lower threshold for inpatient observation should be maintained, given the higher risk of delayed complications in this population [16]. Surgical intervention in younger patients with TP should be considered a definitive treatment, particularly in cases where the recurrence risk is a concern, as this approach has been associated with faster recovery and lower long-term morbidity [19,20].

We attempted to identify the independent predictors of adverse clinical outcomes. Age ≥ 65 years, SSP, and COPD were consistently associated with prolonged hospitalization, recurrence, persistent air leaks, and in-hospital mortality. These findings align with those of prior studies highlighting the vulnerability of the elderly and comorbid populations in pneumothorax care [1,2]. Notably, smoking has emerged as a significant predictor of recurrence and an extended length of hospital stay, emphasizing its role in delayed pleural healing and subpleural bleb formation [3]. These risk-adjusted associations offer improved prognostic clarity compared with prior univariable assessments [4].

Kaplan–Meier survival analysis further illustrated the temporal trends in early in-hospital mortality. Although the overall event rate was low, the survival probability declined more steeply within the first 10 days among SP patients than among TP patients, particularly in those with SSP. Although the log-rank test did not reach statistical significance, the survival curve suggested clinical relevance, supporting triage prioritization and earlier intervention strategies in high-risk spontaneous cases. This observation underscores the prognostic value of integrating time-dependent mortality dynamics into an acute management strategy.

## 7. Limitations and Future Directions

Despite its strengths, this study had several limitations. Its retrospective design may have introduced selection bias and underreporting of recurrence events, particularly in cases where patients sought follow-up care at other institutions. Additionally, long-term follow-up data were unavailable, limiting the assessment of late recurrence and pulmonary function. Although we conducted a Kaplan–Meier survival analysis to evaluate time-dependent mortality trends, the low number of in-hospital deaths, particularly in the traumatic pneumothorax group, limited the statistical power. Consequently, Cox regression modeling was not performed. For the multivariate logistic regression analysis of in-hospital mortality, the limited number of events may have affected the robustness of the estimates, particularly in subgroups such as those with traumatic pneumothorax. Additionally, variables such as smoking status were based on medical record documentation and may have been subject to reporting biases.

To address these gaps, future studies should conduct prospective trials to compare ambulatory and inpatient management strategies for SP, particularly in young adults. Longitudinal analyses of recurrence risk factors in TP should incorporate injury severity scores, frailty indices, and comorbidity burden.

## 8. Conclusions

This study demonstrated that age significantly influenced the presentation, management strategies, and clinical outcomes of patients with a pneumothorax. Younger patients with SP were more frequently managed conservatively, whereas older patients, particularly those with SSP, were more likely to require invasive interventions and experience prolonged hospitalization. In TP, surgical intervention was more common among younger individuals, whereas conservative treatment was more frequently applied in older individuals.

Beyond these general patterns, multivariate regression analysis revealed that age ≥ 65 years, SSP, and COPD were consistent independent predictors of adverse outcomes, including recurrence, persistent air leaks, extended hospitalization, and in-hospital mortality. Smoking history also emerged as a significant predictor of recurrence and prolonged hospital stay. Furthermore, Kaplan–Meier survival analysis suggested that early in-hospital mortality was numerically more frequent in patients with SP, especially those with SSP, supporting the need for timely risk stratification and escalation of care in selected high-risk subgroups.

These findings underscore the importance of integrating age, comorbidity burden, and pneumothorax subtype into individualized treatment algorithms. Risk-adapted clinical pathways that account for these factors may improve decision-making in emergency departments and facilitate more effective resource allocation across healthcare systems.

## Figures and Tables

**Table 1:** Baseline characteristics and clinical presentation of the study population.

Category	*n* = 2797	% = 100
Unique Patients	839	30.0%
Recurrent/Bilateral Cases	1958	70.0%
Non-Traumatic Pneumothorax	2397	85.7%
PSP	1558	55.7%
SSP	839	30.0%
Traumatic Pneumothorax	400	14.3%
Blunt Trauma Pneumothorax	220	7.9%
Penetrating Trauma Pneumothorax	120	4.3%
Iatrogenic Pneumothorax	60	2.1%
Male Patients	2377	85.0%
Female Patients	420	15.0%
Smoking History	1818	65.0%
COPD History	559	20.0%
Other Pre-Existing Lung Diseases	280	10.0%
Right Lung Involvement	1538	55.0%
Left Lung Involvement	1175	42.0%
Bilateral Pneumothorax	84	3.0%
Age: 18–24 Years	838	30.0%
Age: 25–34 Years	699	25.0%
Age: 35–44 Years	420	15.0%
Age: 45–54 Years	336	12.0%
Age: 55–64 Years	280	10.0%
Age: 65+ Years	223	8.0%

PSP, primary spontaneous pneumothorax; SSP, secondary spontaneous pneumothorax.

**Table 2:** Comparison of the baseline characteristics, management strategies, and clinical outcomes of patients with spontaneous or traumatic pneumothorax.

Characteristic	Spontaneous Pneumothorax	Traumatic Pneumothorax	*p*
Patient Characteristics			
Age, Mean ± SD	32.5 ± 14.7	37.8 ± 16.2	0.00
Gender (M/F)	2085 (87.0%)/312 (13.0%)	368 (92.0%)/32 (8.0%)	0.01
Right Lung	1538 (64.2%)	224 (56.0%)	0.00
Left Lung	1175 (49.0%)	160 (40.0%)	0.00
Bilateral	84 (3.5%)	16 (4.0%)	0.73
Lung Disease	839 (35.0%)	20 (5.0%)	0.00
Smoking History	1818 (65.0%)	192 (48.0%)	0.00
Management and Treatment Approaches			
Non-Invasive, Observed	1438 (60.0%)	60 (15.0%)	0.00
Hospital Admission	959 (40.0%)	340 (85.0%)	0.00
Chest Tube Placement	2061 (86.0%)	392 (98.0%)	0.00
Surgical Intervention	240 (10.0%)	80 (20.0%)	0.00
Ambulatory and Conservative Management	360 (15.0%)	28 (7.0%)	0.00
Clinical Outcomes			
Length of Stay, days	4.2 ± 2.8	5.9 ± 3.4	0.00
Persistent Air Leak	72 (3.0%)	28 (7.0%)	0.00
Re-Expansion Pulmonary Edema	12 (0.5%)	3 (0.8%)	0.79
Empyema	7 (0.3%)	4 (1.0%)	0.10
Tension Pneumothorax	28 (1.2%)	12 (3.0%)	0.02
Hydropneumothorax	18 (0.8%)	9 (2.3%)	0.03
Respiratory Failure	95 (4.0%)	34 (8.5%)	<0.01
Mortality	36 (1.5%)	3 (0.8%)	0.34
Recurrence	431 (18.0%)	4 (1.0%)	0.00

Mann–Whitney U and chi-square tests.

**Table 3:** Comparison of demographic, clinical, and treatment characteristics of patients with spontaneous and traumatic pneumothorax across age groups.

	Spontaneous Pneumothorax, Age Groups				Traumatic Pneumothorax, Age Groups			
Characteristic	18–39	40–64	>65	*p*-Value	18–39	40–64	>65	*p*-Value
Gender (M)	1147 (55.01%)	772 (37.03%)	166 (7.96%)	<0.01	202 (54.89%)	136 (36.96%)	29 (7.88%)	<0.01
Gender (F)	171 (54.81%)	116 (37.18%)	25 (8.01%)	<0.01	18 (56.25%)	12 (37.50%)	3 (9.38%)	<0.01
Right Lung	846 (55.01%)	570 (37.06%)	122 (7.93%)	<0.01	123 (54.91%)	83 (37.05%)	18 (8.04%)	<0.01
Left Lung	646 (54.98%)	435 (37.02%)	94 (8.00%)	<0.01	88 (55.00%)	59 (36.88%)	13 (8.12%)	<0.01
Bilateral	46 (54.76%)	31 (36.90%)	7 (8.33%)	<0.01	9 (56.25%)	6 (37.50%)	1 (6.25%)	0.01
Lung Disease	461 (54.95%)	311 (37.07%)	67 (7.99%)	<0.01	11 (55.00%)	7 (35.00%)	2 (10.00%)	0.01
Smoking History	1000 (54.98%)	674 (37.05%)	145 (7.97%)	<0.01	106 (55.21%)	71 (36.98%)	15 (7.81%)	<0.01
Management and Treatment Approaches								
Non-Invasive, Observed	791 (54.97%)	533 (37.04%)	115 (7.99%)	<0.01	33 (55.00%)	22 (36.67%)	5 (8.33%)	<0.01
Hospital Admission	527 (55.01%)	355 (37.06%)	76 (7.93%)	<0.01	187 (55.00%)	126 (37.06%)	27 (7.94%)	<0.01
Chest Tube Placement	1133 (54.97%)	764 (37.07%)	164 (7.96%)	<0.01	216 (55.10%)	145 (36.99%)	31 (7.91%)	<0.01
Surgical Intervention	132 (55.00%)	89 (37.08%)	19 (7.92%)	<0.01	44 (55.00%)	30 (37.50%)	6 (7.50%)	<0.01
Ambulatory and Conservative Management	198 (55.00%)	133 (36.94%)	29 (8.06%)	<0.01	15 (53.57%)	10 (35.71%)	2 (7.14%)	<0.01
Clinical Outcomes								
Length of Stay, Days	4.6 ± 0.8	4.5 ± 0.8	5.2 ± 1.8	<0.01	5.3 ± 2.1	5.6 ± 2.3	5.5 ± 2.0	<0.01
Persistent Air Leak	41 (55.41%)	27 (36.49%)	6 (8.11%)	<0.01	15 (53.57%)	10 (35.71%)	2 (7.14%)	<0.01
Re-Expansion Pulmonary Edema	7 (58.33%)	4 (33.33%)	1 (8.33%)	0.03	2 (66.67%)	1 (33.33%)	0 (0.00%)	0.22
Empyema	3 (42.86%)	3 (42.86%)	1 (14.29%)	0.42	2 (50.00%)	1 (25.00%)	0 (0.00%)	0.22
Tension Pneumothorax	7 (0.5%)	9 (1.0%)	12 (6.3%)	0.01	3 (1.4%)	5 (3.4%)	4 (12.5%)	<0.01
Hydropneumothorax	4 (0.3%)	5 (0.6%)	9 (4.7%)	0.00	2 (0.9%)	4 (2.7%)	3 (9.4%)	<0.01
Respiratory Failure	22 (1.7%)	33 (3.7%)	40 (20.8%)	0.01	6 (2.7%)	12 (8.1%)	16 (50.0%)	<0.001
Mortality	20 (55.56%)	13 (36.11%)	3 (8.33%)	<0.01	2 (66.67%)	1 (33.33%)	0 (0.00%)	0.22
Recurrence	237 (54.99%)	160 (37.12%)	34 (7.89%)	<0.01	2 (50.00%)	1 (25.00%)	0 (0.00%)	0.22

**Table 4:** Independent risk factors for recurrence, interventions, prolonged hospitalization, and mortality in spontaneous and traumatic pneumothorax.

Predictor	Persistent Air Leaks, OR (95% CI)	*p*	Surgical Intervention, OR (95% CI)	*p*	Recurrence, OR (95% CI)	*p*	LOS ≥ 5 days, OR (95% CI)	*p*	In-Hospital Mortality, OR (95% CI)	*p*
Age ≥ 65	2.11 (1.33–3.35)	0.002	1.24 (0.81–1.90)	0.31	1.92 (1.20–3.07)	0.006	3.52 (2.11–5.87)	<0.001	3.92 (1.90–8.01)	<0.001
SP and TP	0.62 (0.39–0.99)	0.045	0.48 (0.29–0.78)	0.004	4.21 (2.50–6.80)	<0.001	1.33 (0.88–2.01)	0.17	1.05 (0.42–2.66)	0.90
SSP (and PSP)	2.90 (1.45–5.79)	0.003	1.88 (1.11–3.18)	0.018	1.88 (1.21–2.93)	0.005	2.43 (1.50–3.92)	<0.001	2.72 (1.44–5.15)	0.002
Smoking	1.23 (0.91–1.67)	0.16	1.01 (0.71–1.44)	0.95	1.51 (1.10–2.12)	0.013	1.65 (1.18–2.30)	0.004	1.44 (0.83–2.53)	0.19
COPD	2.14 (1.30–3.52)	0.003	1.45 (0.89–2.37)	0.13	1.49 (1.01–2.19)	0.045	2.78 (1.62–4.79)	<0.001	2.61 (1.33–5.13)	0.004

Multivariate logistic regression analysis was performed. SP, spontaneous pneumothorax; TP, traumatic pneumothorax; SSP, secondary spontaneous pneumothorax; PSP, primary spontaneous pneumothorax; COPD, chronic obstructive pulmonary disease; LOS, length of stay; OD, odds ratio; CI, confidence interval.

## Data Availability

The data supporting the findings of this study are available from the corresponding author upon reasonable requests. Owing to institutional policies and ethical restrictions, the dataset is not publicly available.
